# Postoperative Vision-Related Quality of Life in Macula-Off Rhegmatogenous Retinal Detachment Patients and Its Relation to Visual Function

**DOI:** 10.1371/journal.pone.0114489

**Published:** 2014-12-02

**Authors:** Mathijs A. J. van de Put, Lisette Hoeksema, Wouter Wanders, Ilja M. Nolte, Johanna M. M. Hooymans, Leonoor I. Los

**Affiliations:** 1 Department of Ophthalmology, University of Groningen, University Medical Center Groningen, Groningen, the Netherlands; 2 Department of Epidemiology, University of Groningen, University Medical Center Groningen, Groningen, the Netherlands; 3 W.J. Kolff Institute, Graduate School of Medical Sciences, University of Groningen, Groningen, the Netherlands; Charité University Medicine Berlin, Germany

## Abstract

**Objective:**

To determine the vision-related quality of life (VR-QOL) after surgery for macula-off rhegmatogenous retinal detachment (RRD) in relation to visual acuity, contrast acuity, and color vision.

**Methods:**

In a prospective observational study, we included 55 patients with a macula-off RRD. Best corrected visual acuity (BCVA), color vision (saturated and desaturated color confusion indices (CCI)) and contrast acuity were measured at 12 months postoperatively in both the RRD eye and the fellow control eye, and the 25-item National Eye Institute Visual Function Questionnaire (NEI VFQ-25) was filled out.

**Results:**

Operated and fellow control eyes differed significantly in mean LogMAR BCVA (P<0.0001), median Log contrast acuity (P<0.0001), saturated CCI (P = 0.009), and desaturated CCI (P = 0.016). Significant correlations were observed between the NEI VFQ-25 overall composite score and postoperative LogMAR BCVA (R = −0.551, P<0.0001), contrast acuity (R = 0.472, P<0.0001), saturated CCI (R = −0.315, P = 0.023), and desaturated CCI (R = −0.283, P = 0.044).

**Conclusions:**

A lower VR-QOL was highly correlated to a worse postoperative BCVA and contrast acuity and to a lesser extent to color vision disturbances.

## Introduction

Rhegmatogenous retinal detachment (RRD), which refers to a detachment of the neurosensory retina from the underlying retinal pigment epithelium due to a defect in the retina [1], occurs with an incidence of 19/100,000 people/year in the Netherlands [2]. With surgical intervention, the detached neuroretina can be reattached to the retinal pigment epithelium in more than 95% of cases [3]–[5]. In spite of this high anatomic success rate, functional recovery is often compromised [6]–[12], especially when the macula was detached during the RRD, which happens in about 50% of cases [2].

Not only best corrected visual acuity (BCVA) is compromised in these cases, other aspects of central visual function are also compromised after macula-off RRD [11], [13]–[14]. Two modifiable factors are crucial in the recovery of visual function in these cases [4], [8], [15]–[17]. These are the pre-operative duration of the macular detachment (i.e. a longer duration will result in a lower visual acuity (VA), and a worse recovery of color vision) [4], [8], and the preoperative height of the macular detachment (i.e. an increase in height will result in a lower postoperative BCVA) [15]–[17]. Non-modifiable factors influencing the postoperative recovery of visual function include age, refractive error, and preoperative VA [4], [14].

Previous studies identified a strong relation between postoperative visual function and post-operative vision-related quality of life (VR-QOL) as measured by the National Eye Institute Visual Functioning Questionnaire-25 (NEI VFQ-25) in patients operated on for various vitreoretinal disorders, including RRD [18]–[25]. The NEI VFQ-25 ocular composite score and subscores are further explained in the Methods. Zou et al. showed that postoperative quality of life is worse in macula-off compared to macula-on RRD [18]. In Okamoto's study, postoperative BCVA differed significantly between macula-on and macula-off RRD, while scores on the NEI VFQ-25 were similar in both groups of patients [20]. Surprisingly, that study indicated that a worse post-operative contrast acuity was related to a lower score on the NEI VFQ-25 questionnaire, whereas a low post-operative VA was not [20].

We could not find previous studies addressing post-operative quality of life specifically in macula-off RRD patients in relation to BCVA, contrast acuity and color vision. Therefore, the purpose of the present study is to determine the postoperative VR-QOL after macula-off RRD one year after successful reattachment of the retina, and to assess which aspects of postoperative visual function (VA, contrast acuity, or color vision) are most closely related herewith. In addition, we evaluated whether pre-operative, intra-operative, and postoperative factors are associated with a difference in postoperative VR-QOL.

## Methods

### Study design

We conducted a prospective observational study in patients with a first presentation of macula-off RRD who had an attached retina at 12 months after the first surgical procedure. Reattachment was obtained by one or more surgical procedures. The research protocol was approved by the University Medical Center Groningen (UMCG) review board ethics committee, and was carried out in accordance with the tenets of the declaration of Helsinki. The study was registered with the Dutch Trial Register (NTR839). All patients were operated on at the ophthalmology department of the UMCG. The study was carried out over a three-year period (February 1, 2007–February 1, 2010).

### Study population

Adult patients visiting the ophthalmology department of the UMCG with a first presentation of unilateral macula-off RRD of 24 hours to 6 weeks duration were invited to participate in this study. Included in the study were patients of 18 years and older who had given their written informed consent. Patients had to be able to pinpoint their drop in VA to a specific 24-hour period in case of a 24-hour to 1 week macular detachment, and to a period of less than one week in case of a macular detachment of one to six weeks. The cut-off point of ≤1 week or >1 week is conform the available literature [11], [13]. Patients with macular detachment of more than 6 weeks duration were excluded, because they are considered rare, and yield a worse prognosis [9]. Surgery was performed within 24–72 hours after presentation at the ophthalmology department. Excluded were patients with a history of congenital or acquired pathology with an effect on visual function in one or both eyes (with the exception of congenital defects in color vision), or pathology observed at presentation after their macula-off RRD that could influence post-operative VA.

### Preoperative measurements

We acquired the following patients' characteristics: age, gender, affected eye, ophthalmic history and family history for RRD. In addition, we scored the number of retinal quadrants detached at presentation, and the presence, and grade of PVR [26]. Using standardised protocols, the refractive error and BCVA using the Early Treatment of Diabetic Retinopathy Study (ETDRS) chart were determined in the affected and fellow control eye [27]. All VA measurements were converted to logMAR equivalents of ETDRS acuity for analysis. Light perception or hand movements were coded as logMAR VA of 3.0.

Duration and height of macular detachment were determined using the following scoring system. Macula-off RRDs of less than one week duration were scored per day, and of more than one week duration they were scored as 11 days (1–2 weeks duration), 18 days (2–3 weeks duration), 25 days (3–4 weeks duration), 32 days (4–5 weeks duration), and 39 days (5–6 weeks duration), respectively.

To measure the height of the detachment at the position of the central macula by ultrasonography the relative positions of the central macula and the optic nerve head were determined before performing a ultrasonography. For this purpose, digital fundus photographs of both eyes were made using the TRC-50 IX fundus camera (Topcon 9B ltd. UK). On both fundus photographs, the distance between the optic nerve head and fovea was measured using the software package IMAGEnet2000 2.53. The measured distance in the affected eye was used to determine the central position of the macula and at this position the height of the macular detachment was measured by ultrasonography. In those cases (i.e. bullous retinal detachment), in which the measurement of the distance between the macula and optic nerve head could not be performed on the photograph of the affected eye, the measurement of this distance in the fellow eye was used [28]. In each patient, two measurements were made with the patient in an upright position (as this represents the position most patients would have taken for most of the time before presentation during the day) and the average of both measurements was used for further analysis.

### Surgical procedure (intraoperative data)

Based on clinical presentation, patients were either operated on by an external procedure (i.e. encircling band and/or buckle) or by 20 Gauge PPV (with or without an encircling band). In PPV cases, either a short acting tamponade (i.e. sulfur hexafluoride gas (SF6)) or a long acting tamponade was used (i.e. octafluoropropane (C3F8) or silicone oil). Collected data refer to the first surgical procedure in all cases.

### Postoperative measurements

#### Visual function

At 12 months postoperatively, we measured BCVA using the ETDRS chart [27], contrast acuity using the Pelli Robson chart [29], Farnsworth D-15 saturated and Lanthoni desaturated color confusion indexes (CCI) [30]. All measurements were done in the affected and fellow control eye. Information on postoperative success (i.e. primary or secondary) was acquired. Also, the number of surgical procedures needed to obtain an attached retina, were recorded.

#### Quality of life

At 12 months postoperatively, patients were requested to self-administer the validated Dutch version [25] of the NEI VFQ-25 to assess their VR-QOL [21]–[25]. This questionnaire has been developed by the research and development corporation (RAND), and funded by the NEI. The NEI VFQ-25 comprises 25 items that require the patient to assess the influence of visual disability and visual symptoms on generic health domains such as emotional well-being and social functioning, in addition to task-oriented domains related to daily visual functioning. Each item is assigned to one of the following twelve subscales: general health, general vision, ocular pain, near activities, distance activities, vision specific social functioning, vision specific mental health, vision specific role difficulties, vision specific dependency, driving, color vision, and peripheral vision [21]–[25]. Each subscale consists of a minimum of one and a maximum of four items. We used the standard algorithm to calculate the scale scores. The subscales are 0 to 100 points, where 100 indicates the highest possible function or minimal subjective impairment. The NEI VFQ-25 overall composite score (OCS) is calculated as the unweighted average response to all items, excluding the question on general health.

#### Cataract

Because of an increased risk of cataract development after PPV, which may influence postoperative measurements, we scored the level of cataract using the lens opacities classification system III (LOCS III) in both eyes at pre-determined post-operative intervals [31]. In addition, BCVA was assessed at those time points, and in case of a visually significant cataract (n = 26 eyes) a cataract extraction was performed before the 12 months measurement.

### Statistical analyses

Data were analysed using SPSS software package, version 16.0 (Chicago, Illinois, USA). A one-tailed paired Student's t-test (we expect worse visual function in the operated eye) or Wilcoxon signed rank test was used to explore statistical differences in visual function parameters between operated and fellow control eyes depending on the distribution of the variable. Spearman's correlation coefficients were calculated to explore significant correlations between the different postoperative visual function parameters. The relationships between age, preoperative factors, postoperative visual function tests (LogMAR VA, Log contrast acuity, saturated,and desaturated CCI) and the NEI VFQ-25 scores were examined using Spearman's correlation coefficients. To determine differences in NEI VFQ-25 OCS and subscores in subgroups we used a Mann-Whitney U test in case of two groups or a Kruskal-Wallis test in case of more than two groups. In the latter case post-hoc analyses were performed for pairwise comparisons between subgroups. All tests were considered statistically significant at a p-value of less than 0.05, except for the Kruskal-Wallis post-hoc analysis, for which a significance threshold of 0.05 divided by the number of groups was used.

## Results

### Study characteristics

#### RRD-study

A total of 56 patients gave their written informed consent and were included. In 46 patients retinal re-attachment was obtained after one surgical procedure, whereas ten patients had one or more re-detachments. One patient died during the study period and was therefore excluded from analysis. In all remaining 55 patients, the retina was still surgically attached twelve months after the initial surgical procedure. Missing data further consisted of: visual function tests at 12 months (n = 1), saturated (n = 2) and desaturated (n = 3) CCI, because of color blindness (n = 2) and unknown reasons (n = 1).

Table 1 summarizes the preoperative data on general patient characteristics and type of surgery. Briefly, the mean age was 60.4 years, more male than female patients were included (2.7∶1), right and left eyes were equally involved, and most eyes were phakic (67.3%). A PPV was most frequently chosen as the primary surgical procedure (n = 45 (81.8%)). This was combined with an encircling band in about half the cases (n = 27). Data on refractive error could reliably be obtained in phakic eyes (n = 37). In pseudophakic patients, data on refractive error prior to cataract extraction were not available in 18 eyes. These were coded as missing data. In case of known refractive error, no significant associations with visual function or NEI VFQ-25 scores were observed.

**Table 1:** Preoperative patient characteristics, lens status, and type of surgery.

Characteristics	Number (%)	Mean age ± SD	Scleral buckling/PPV (%)
Total	55 (100.0)	60.4±11.2	10 (18.2)/45 (81.8)
Male	40 (72.7)	61.4±9.8	5 (12.5)/35 (87.5)
Female	15 (27.3)	57.8±14.4	5 (33.3)/10 (66.7)
Phakic	37 (67.3)	59.5±8.3	10 (27.0)/27 (73.0)
Pseudophakic	18 (32.7)	62.2±15.8	0 (0.0)/18 (100.0)

SD: Standard deviation, PPV: Pars plana vitrectomy.

Postoperative BCVA, Log contrast acuity, saturated and desaturated CCI in operated eyes were significantly worse than in fellow control eyes (Table 2). We observed high correlations between postoperative LogMAR BCVA, log contrast acuity, saturated, and desaturated CCI's (Table 3).

**Table 2:** Visual function tests in operated versus in fellow control eyes.

Visual acuity	Number	Operated eye, Mean ± SD	Fellow eye, Mean ± SD	P-value
Preoperative visual acuity (LogMAR)	55	2.15±1.10	0.09±0.20	<0.0001
Preoperative visual acuity (Snellen)^a^		HM	16/20	
Postoperative visual acuity (LogMAR)	54	0.35±0.37	0.05±0.11	<0.0001
Postoperative visual acuity (Snellen)^a^		4/10–5/10	20/20	

Visual function	Number	Operated eye, Median (ranges)	Fellow eye, Median (ranges)	P-value
Postoperative contrast acuity (Log)	54	1.45 (0.00–1.70)	1.55 (1.20–1.90)	<0.0001
Postoperative color vision saturated CCI	52	1.16 (1.00–3.09)	1.00 (1.00–2.59)	0.009
Postoperative color vision desaturated CCI	51/51	1.77 (1.00–3.20)	1.52 (1.00–2.59)	0.016

BCVA: best corrected visual acuity, CCI: Color Confusion Indices, SD: standard deviation, HM: hand movements. Postoperative measurements were performed 12 months after retinal detachment surgery.

a Mean LogMAR visual acuity converted to Snellen visual acuity.

**Table 3:** Spearman's rank correlation coefficients between LogMAR BCVA, log contrast acuity, and CCI (saturated and desaturated).

	*R*	*P-value*
LogMAR BCVA/contrast acuity	−0.633	<0.0001
LogMAR BCVA/saturated CCI	0.556	<0.0001
LogMAR BCVA/desaturated CCI	0.446	0.001
Contrast acuity/saturated CCI	−0.415	0.002
Contrast acuity/desaturated CCI	−0.393	0.004
Saturated CCI/desaturated CCI	0.734	<0.0001

BCVA: best corrected visual acuity, CCI: Color vision Confusion Index. Measurements were performed 12 months after retinal detachment surgery.

### Quality of life

Table 4 presents the outcomes of the NEI VFQ-25 scores in relation to demographic, patient, and surgical parameters. Overall, scores are relatively high when compared to previous studies on macula-on and macula-off RRD, epiretinal membrane (ERM) and macular hole (MH) (Table 5). Only limited differences between subgroups of patients were observed.

**Table 4:** NEI VFQ-25 overall composite score and subscale scores (mean ± SD).

		GH	GV	OP	NA	DA	VSSF	VSMH	VSRD	VSD	D	CV	PV	OCS^a^
		(n = 55)	(n = 55)	(n = 55)	(n = 55)	(n = 54)	(n = 55)	(n = 55)	(n = 55)	(n = 55)	(n = 45)	(n = 50)	(n = 53)	(n = 55)
*Total group*	(n = 55)	60.5±21.3	71.3±12.0	88.4±15.2	85.6±13.8	86.1±12.6	98.4±4.8	87.6±14.7	88.2±16.2	98.3±5.4	84.2±17.1	97.0±13.0	91.0±16.3	88.9±7.9
*Gender*	- Male (n = 40)	61.9±20.4	71.0±20.4	87.2±16.6	85.6±13.3	86.8±11.6	97.8±5.6	87.7±15.1	87.2±15.9	97.9±6.2	87.1±15.4	96.4±15.0	91.5±16.7	88.4±84
	- Female (n = 15)	56.7±24.3	72.0±12.6	91.7±10.2	85.6±15.6	83.9±15.5	100.0±0.0	87.5±14.0	90.8±17.3	99.4±2.2	75.0±19.7	98.3±6.5	90.0±15.8	89.8±6.8
	P-value	0.256	0.907	0.546	0.861	0.679	0.116	0.899	0.314	0.396	0.052	0.939	0.574	0.777
*Family* b	- Positive (n = 6)	58.3±25.8	63.3±15.1	93.8±10.5	77.8±16.4	81.9±6.3	97.9±5.1	83.3±10.9	87.5±15.8	100.0±0.0	83.3±18.0	100.0±0.0	83.4±25.8	85.24±9.0
	- Negative (n = 49)	60.7±21.0	72.2±11.4	87.8±15.6	86.6±13.4	86.6±13.2	98.5±4.9	88.1±15.1	88.3±16.4	98.1±5.7	84.3±17.3	96.6±13.8	92.0±14.8	89.18±7.8
	P-value	0.755	0.148	0.380	0.166	0.182	0.653	0.148	0.788	0.327	0.854	0.514	0.467	0.19
*Lens (pre-op)*	- Phakic (n = 37)	62.2±21.7	70.3±10.1	87.8±16.5	84.2±12.8	85.7±12.2	98.7±4.9	84.8±16.0	88.2±16.4	97.8±6.4	82.8±18.2	97.7±9.6	90.0±16.3	88.2±7.8
	- Pseudophakic (n = 18)	56.9±20.7	73.3±15.3	89.6±12.3	88.4±15.7	86.9±13.9	97.9±4.8	93.4±9.5	88.2±16.3	99.5±2.0	86.9±15.0	95.6±18.2	93.1±16.7	89.9±8.3
	P-value	0.376	0.390	0.907	0.138	0.530	0.370	**0.012**	0.960	0.258	0.451	0.980	0.331	0.212
*Quadrants* c	- 1–2 (n = 35)	58.6±19.1	72.0±11.1	91.4±13.5	87.6±12.7	86.9±11.8	99.3±2.9	90.6±9.6	92.1±13.6	98.8±3.6	85.4±18.1	96.9±13.8	90.4±17.4	90.0±6.7
	- 3–4 (n = 19)	61.8±24.1	69.5±13.9	82.2±16.8	81.6±15.6	84.6±14.5	96.7±7.0	82.9±20.6	80.3±18.3	97.4±7.8	81.6±15.7	97.1±12.1	91.7±14.9	86.4±9.8
	P-value	0.733	0.481	**0.017**	0.163	0.616	0.084	0.181	**0.011**	0.630	0.288	0.960	0.970	0.319
*PVR*	- Grade A (n = 30)	59.2±22.2	72.7±11.1	90.8±12.3	87.2±13.8	88.8±10.6	99.2±3.2	91.0±8.6	88.3±14.6	98.6±3.8	86.3±13.3	96.3±15.0	90.0±18.1	89.6±6.5
	- Grade B (n = 16)	60.9±15.7	71.3±14.5	85.2±16.6	85.4±15.4	86.1±9.8	96.9±7.2	87.9±11.7	88.3±18.5	99.5±2.1	80.8±22.2	96.4±13.4	90.0±15.8	88.1±9.0
	- Grade C (n = 8)	59.4±26.5	65.0±9.3	84.4±21.9	79.2±10.9	76.0±20.1	98.4±4.4	75.8±28.8	85.9±19.4	94.8±11.7	81.3±26.7	100.0±0.0	96.4±9.4	86.4±11.2
	P-value	0.866	0.235	0.486	0.227	0.159	0.440	0.347	0.848	0.400	0.955	0.737	0.650	0.899
*Duration* d	- ≤1 week (n = 29)	60.3±19.5	70.3±11.5	89.66±15.3	86.2±13.4	86.2±12.2	99.1±4.6	90.1±10.8	88.4±14.1	98.3±4.01	84.1±17.6	95.4±17.0	92.9±13.4	89.2±7.1
	-> 1 week (n = 26)	60.6±23.6	72.3±12.7	87.02±15.2	84.9±14.5	86.0±13.3	97.6±5.0	84.9±18.0	88.0±15.5	98.4±6.7	84.3±16.9	98.9±5.2	89.0±19.2	88.2±8.9
	P-value	0.875	0.746	0.338	0.815	0.874	0.075	0.244	0.708	0.322	0.934	0.620	0.628	0.781
*Success rate*	- Primary (n = 45)	58.9±21.4	72.0±12.4	89.7±13.1	87.2±13.8	87.0±12.2	98.6±4.8	89.9±10.9	88.1±15.1	98.7±3.5	87.2±12.9	97.0±13.8	92.1±15.0	89.9±6.7
	- Secondary (n = 10)	67.5±20.6	68.0±10.3	82.5±22.2	78.3±11.9	81.5±14.3	97.5±5.3	77.5±24.0	88.8±21.6	96.7±10.5	67.9±27.4	96.9±8.8	86.1±22.0	83.3±10.9
	P-value	0.229	0.313	0.104	**0.039**	0.255	0.333	0.072	0.506	0.865	0.085	0.440	0.480	0.053
*Re-detached* e	- 1 (n = 7)	64.3±19.7	68.6±10.7	94.6±6.7	79.8±14.3	81.9±14.4	98.2±4.7	88.4±10.5	91.1±23.6	100.0±0.0	75.0±27.6	95.0±11.2	92.9±18.9	88.1±8.0
	- 2 (n = 3)	75.0±25	66.7±11.5	54.2±19.1	75.0±0	80.6±17.3	95.8±7.2	52.1±29.5	83.3±19.1	88.9±19.2	50.0±23.6	100.0±0.0	62.5±17.7	72.0±8.8
	P-value	0.458	0.789	**0.012**	0.479	0.794	0.513	**0.027**	0.207	0.127	0.241	0.439	0.054	0.053
*Technique*	- Conventional (n = 10)	62.5±21.2	76.0±8.4	90.0±16.5	90.8±10	87.5±11.9	100.0±0.0	90.0±12.2	98.8±4.0	98.3±3.5	85.4±24.3	100.0±0.0	92.5±12.1	91.8±6.0
	- PPV (n = 45)	60.0±21.6	70.2±12.5	88.1±15.1	84.4±14.4	85.7±12.9	98.1±5.3	87.1±15.3	85.8±17	98.3±5.8	83.9±15.6	96.3±14.3	90.7±17.3	88.1±8.2
	P-value	0.847	0.121	0.554	0.235	0.856	0.226	0.472	**0.014**	0.497	0.322	0.408	0.953	0.209
*TPPV & Band* f	- Band (n = 18)	61.1±21.4	74.4±15.0	89.6±12.3	87.0±16.5	89.8±10.5	98.6±4.0	92.0±10.2	88.2±15.1	99.5±2.0	86.5±14.6	100.0±0.0	91.7±17.1	90.3±7.9
	- No band (n = 27)	59.3±22.1	67.4±9.8	87.0±16.8	82.7±12.9	83.2±13.8	97.7±6.0	83.8±17.3	84.3±18.2	97.5±7.2	81.9±16.4	93.8±18.4	90.0±17.7	86.6±8.3
	P-value	0.890	0.056	0.755	0.169	0.099	0.694	**0.038**	0.518	0.318	0.372	0.135	0.699	0.073
*Tamponade* g	- Short acting (n = 35)	59.3±21.1	72.6±12.0	91.8±10.9	87.1±13.5	85.6±18.6	98.6±4.0	91.6±8.2	86.4±16.4	99.1±3.4	84.8±20.1	96.9±13.8	91.2±17.3	89.8±6.7.7
	- Long acting (n = 10)	62.5±24.3	62.0±11.4	75.0±20.4	75.0±14.2	77.5±15.2	96.3±8.4	71.3±23.0	83.8±19.6	95.8±10.6	65.3±15.3	94.4±16.7	88.9±18.2	81.9±10.2
	P-value	0.788	**0.041**	**0.012**	**0.021**	0.061	0.657	**<0.001**	0.778	0.581	**0.004**	0.841	0.714	**0.018**
*Lens (post-op)*	- Phakic (n = 11)	59.1±16.9	74.6±9.3	85.2±21.5	92.4±8.7	87.1±12.0	97.7±7.5	90.3±11.0	90.9±15.9	98.5±3.4	81.3±23.0	95.5±12.6	90.9±12.6	89.8±8.6
	- Pseudophakic (n = 42)	61.3±22.2	71.0±12.7	89.6±13.5	84.9±13.9	86.9±11.8	98.8±3.7	88.2±14.2	87.2±16.7	98.2±6.0	84.8±15.9	97.4±16.2	92.5±16.2	88.6±7.8
	P-value	0.779	0.315	0.981	0.117	0.927	0.965	0.626	0.487	0.641	0.873	0.645	0.387	0.483

Nominal significant values are indicated in bold.

NEI VFQ-25: National Eye Institute Visual Functioning Questionnaire-25, SD: standard deviation, GH: General Health, GV: General vision, OP: Ocular pain, NA: Near activities, DA: Distance activities, VSSF: Vision Specific Social Functioning, VSMH: Vision Specific Mental Health, VSRD: Vision Specific Role Difficulties, VSD: Vision Specific Dependency, D: Driving, CV: Color Vision, PV: Peripheral Vision, OCS: Overall Composite Score, PVR: Proliferative vitreoretinopathy, PPV: Pars Plana Vitrectomy.

a Average of vision-targeted subscale scores, without GH.

b Family history of RRD.

c Numbers of detached retinal quadrants at presentation.

d Duration of macular detachment.

e Number of re-detachments.

f PPV with or without an encircling band.

g Postoperative tamponade after PPV; SF6 gas (short acting) versus C3F8 gas & silicon oil (long acting).

**Table 5:** NEI VFQ-25 overall composite score and subscale scores for the present study and previously published studies, mean (SD).

Study	Group composition	Age	GH	GV	OP	NA	DA	VSSF	VSMH	VSRD	VSD	D	CV	PV	OCS
Van de Put	RRD mac-off	60.4 (11.2)	60.5	71.3	88.4	85.6	86.1	98.4	87.6	88.2	98.3	84.2	97.0	91.0	88.9
n = 55	12 months		(21.3)	(12.0)	(15.2)	(13.8)	(12.6)	(4.8)	(14.7)	(16.2)	(5.4)	(17.1)	(13.0)	(16.3)	(7.9)
Okamoto [18]	RRD mac-on & off	52.3 (13.2)	54.2	70.7	82.4	75.3	75.6	88.2	77.5	78.5	87.2	75.5	94.0	72.2	79.6
n = 55	3 months		(16.6)	(14.9)	(14.7)	(17.3)	(16.9)	(14.9)	(18.4)	(24.3)	(17.3)	(24.0)	(10.8)	(21.0)	(14.2)
Okamoto [19]	RRD mac-on & off	51.9 (13.8)	54.4	71.3	83.3	76.6	76.3	89.2	78.2	79.2	88.1	77.0	94.1	72.5	80.3
n = 51	6 months		(17.1)	(13.4)	(13.2)	(16.1)	(15.8)	(13.5)	(17.2)	(22.0)	(15.3)	(22.0)	(10.7)	(20.2)	(12.5)
Schweitzer [32]	Acute PVD	Females: 62.1 (7.6)	80.6	85.8	89.6	89.6	94.4	99.1	91.8	95.7	99.4	87.9	99.1	95.5	93.5
n = 84	6 weeks	Males: 64.5 (6.6)	(16.0)	(10.9)	(12.9)	(10.9)	(8.3)	(3.4)	(9.8)	(8.6)	(3.0)	(14.6)	(6.1)	(11.1)	(6.2)
Okamoto [19]	MH	64.3 (9.6)	53.6	69.0	84.8	70.2	72.2	82.7	76.6	78.6	85.7	78.6	85.7	75.8	79.2
n = 42	3 months		(17.1)	(14.1)	(16.0)	(18.8)	(19.4)	(15.6)	(14.1)	(21.8)	(19.2)	(12.6)	(19.2)	(19.3)	(13.0)
Hirneiss [33]	MH	67 (-)	61.1	62.6	88.0	71.3	80.0	84.7	88.5	71.1	88.7	66.8	92.1	85.8	79.1
n = 59	3 months		(18.4)	(21.6)	(18.0)	(20.4)	(20.1)	(18.6)	(19.8)	(28.2)	(18.7)	(30.5)	(10.4)	(20.5)	(15.4)
Tranos [34]	MH	70 (9)	64.2	70.5	84.1	77.2	81.1	92.1	74.7	74.5	88.8	82.6	95.8	88.3	82.4
n = 26	4 months		(18.2)	(14.5)	(20.4)	(22.8)	(20.8)	(17.5)	(27.4)	(26.6)	(19.5)	(29.3)	(13.2)	(20.1)	(14.1)
Okamoto [19]	ERM	67 (8.4)	55.3	69.1	87.9	71.0	73.4	83.0	78.4	76.5	88.9	76.0	89.4	72.7	78.5
n = 33	3 months		(15.0)	(11.3)	(11.9)	(18.2)	(16.0)	(9.3)	(13.1)	(20.0)	(11.0)	(16.9)	(12.5)	(17.0)	(8.4)
Ghazi [35]	ERM	66 (13)	65.8	70.5	86.8	74.6	79.4	94.7	79.5	80.6	91.2	88.0	92.1	86.1	83.3
n = 20	4 months		(27.9)	(12.2)	(18.4)	(24.4)	(18.3)	(12.7)	(22.7)	(26.5)	(17.0)	(8.4)	(11.9)	(23.0)	(15.5)
Matsuoka [36]	ERM	70 (9)	61	68	84	78	77	85	78	85	90	71	89	71	81
n = 26	12 months		(2)	(3)	(3)	(2)	(3)	(2)	(4)	(3)	(3)	(4)	(3)	(4)	(2)

VFQ-25: National Eye Institute Visual Functioning Questionnaire-25, GH: General Health, GV: General vision, OP: Ocular pain, NA: Near activities, DA: Distance activities, VSSF: Vision Specific Social Functioning, VSMH: Vision Specific Mental Health, VSRD: Vision Specific Role Difficulties, VSD: Vision Specific Dependency, D: Driving, CV: Color Vision, PV: Peripheral Vision, OCS: Overall Composite Score, SD: standard deviation, RRD: rhegmatogenous retinal detachment, PVD: posterior vitreous detachment, MH: macular hole, ERM: epiretinal membrane.

In summary, even though only small differences existed between vision related quality of life OCS and subscale scores, lower scores on vision related quality of life may be related to more extensive surgery, long-term intraocular tamponades and re-detachment. Patients with a more extensive retinal detachment had lower scores on the subscales ocular pain (i.e. experienced more ocular pain) and vision specific role difficulties. Primary surgical success was associated with higher scores on the subscale near activities and OCS. A higher number of re-detachment surgeries was associated with lower scores on ocular pain (i.e. more pain) and vision specific mental health. Patients operated on by PPV had lower scores on vision specific role difficulties, and patients in whom a PPV was combined with an encircling band scored higher on vision specific mental health. Patients receiving a shorter acting gas tamponade (SF6) instead of longer acting gas tamponade (C3F8) or silicone oil were observed to have higher scores on general vision, ocular pain, near activities, vision specific mental health, driving and on the OCS. Preoperatively pseudophakic patients (RRD eye or fellow eye) had higher scores on the vision specific mental health subscale than phakic patients.

Table 6 provides information on the Spearman's correlation coefficients of age, preoperative factors, and postoperative visual function tests and NEI VFQ-25 outcomes. In general, worse outcomes of visual function tests are correlated with lower NEI VFQ-25 scores. Correlations between BCVA or contrast acuity and NEI VFQ-25 scores were more numerous and stronger than those between color vision and NEI VFQ-25 scores.

**Table 6:** Spearman's rank correlation coefficients between age, preoperative factors (height and duration of retinal detachment), LogMAR BCVA, contrast acuity, CCI (saturated and desaturated), and NEI VFQ-25 subscale scores and OCS.

	GH	GV	OP	NA	DA	VSSF	VSMH	VSRD	VSD	D	CV	PV	OCS
						R (P-value)							
Age (years) (n = 55)	−0.099	−0.098	0.136	−0.005	−0.186	−0.225	0.109	−0.151	−0.156	0.028	−0.246	−0.117	−0.184
	(0.473)	(0.479)	(0.323)	(0.969)	(0.179)	(0.098)	(0.427)	(0.272)	(0.256)	(0.857)	(0.086)	(0.403)	(0.178)
Height^a^ (upright) (n = 55)	−0.151	−0.043	−0.08	−0.238	−0.148	−0.189	0.03	−0.204	−0.025	−0.177	−0.121	−0.22	−0.103
	(0.271)	(0.753)	(0.562)	(0.08)	(0.287)	(0.167)	(0.827)	(0.136)	(0.854)	(0.244)	(0.402)	(0.113)	(0.456)
Duration^b^ (n = 29)	0.320	0.253	−0.016	−0.127	−0.088	0.082	−0.064	0.070	−0.033	0.003	0.279	−0.124	0.081
≤7 days	(0.090)	(0.186)	(0.935)	(0.513)	(0.657)	(0.672)	(0.741)	(0.719)	(0.865)	(0.987)	(0.159)	(0.531)	(0.676)
Duration^b^ (n = 25)	0.142	−0.108	−0.035	−0.106	−0.234	−0.165	−0.165	0.195	−0.158	−0.043	0.132	−0.291	0.048
>7 days ≤6 weeks	(0.499)	(0.609)	(0.870)	(0.613)	(0.260)	(0.431)	(0.431)	(0.349)	(0.450)	(0.869)	(0.559)	(0.168)	(0.821)
LogMAR BCVA (n = 54)	−0.196	−0.391	−0.304	−0.517	−0.317	−0.196	−0.405	−0.307	−0.121	−0.588	−0.238	−0.163	−0.551
	(0.155)	**(0.003)**	**(0.026)**	**(<0.0001)**	**(0.021)**	(0.157)	**(0.002)**	**(0.024)**	(0.384)	**(<0.0001)**	(0.099)	(0.243)	**(<0.0001)**
Contrast acuity (n = 54)	0.058	0.394	0.142	0.470	0.349	0.287	0.264	0.277	0.014	0.466	0.281	0.234	0.472
	(0.677)	**(0.003)**	(0.305)	**(<0.0001)**	**(0.010)**	**(0.036)**	(0.053)	**(0.042)**	(0.918)	**(0.001)**	(0.051)	(0.092)	**(<0.0001)**
CCI saturated (n = 52)	−0.129	−0.278	−0.268	−0.233	−0.174	−0.248	−0.259	−0.053	0.044	−0.357	−0.192	−0.142	−0.315
	(0.363)	**(0.046)**	(0.055)	(0.096)	(0.221)	(0.076)	(0.064)	(0.708)	(0.756)	**(0.019)**	(0.197)	(0.320)	**(0.023)**
CCI desaturated (n = 52)	−0.099	−0.207	−0.136	−0.138	−0.162	−0.029	−0.152	−0.140	−0.108	−0.200	−0.265	−0.199	−0.283
	(0.488)	(0.145)	(0.342)	(0.332)	(0.261)	(0.842)	(0.287)	(0.327)	(0.449)	(0.198)	(0.072)	(0.166)	**(0.044)**

Nominal significant values are indicated in bold.

NEI VFQ-25: National Eye Institute Visual Functioning Questionnaire-25, GH: General Health, GV: General vision, OP: Ocular pain, NA: Near activities, DA: Distance activities, VSSF: Vision Specific Social Functioning, VSMH: Vision Specific Mental Health, VSRD: Vision Specific Role Difficulties, VSD: Vision Specific Dependency, D: Driving, CV: Color Vision, PV: Peripheral Vision, OCS: Overall Composite Score. BCVA: best corrected visual acuity, CCI: Color vision Confusion Index.

a Height of the retinal detachment in an upright position.

b Duration of macular detachment.

## Discussion

In our study on patients with macula-off RRD, NEI VFQ-25 OCS and subscale scores were relatively high with a mean OCS of 88.5. The best possible score on each question was 100 and the second best 75 or 80. This means that most patients scored between the best and second best score. Although the scores were somewhat lower than those in a normal working population and in a population with posterior vitreous detachment (PVD) [32], [37], they were higher when compared to other studies addressing NEI VFQ-25 scores in RRD patients (Table 5) [19]–[20]. However, it is difficult to directly compare the results of our study to the results of others, because of differences in study design.

For example, Okamoto et al. [19]–[20] who included macula-on and off patients observed lower NEI VFQ-25 scores than we did [19]–[20]. Possible explanations for this are that they had a shorter follow-up time of 6 months, that cataract may have developed in patients operated on by PPV, and that different methods were used to perform visual function tests. Remarkably, in Okamoto's study, patients were younger and the subgroup of macula-off RRD patients was smaller, whereas that study excluded patient with PVR and re-detachment [19]–[20]. Since these factors are associated with a better postoperative VA, and VA in their study was higher than in ours, one would have expected the NEI VFQ-25 OCS and subscores to be higher in their study [19]–[20].

We observed correlations between NEI VFQ-25 OCS, and subscale scores and postoperative BCVA, contrast acuity and post-operative CCI. All tested postoperative visual function parameters were highly correlated with each other. This suggests that BCVA, contrast acuity, and color vision represent interdependent aspects of macular function. However, our observations suggest that - of all tested variables - postoperative BCVA and contrast acuity in the RRD-eye are the most important determinants of postoperative VR-QOL.

In contrast to postoperative visual functioning, other patient and surgery related aspects showed very few correlations with NEI VFQ-25 outcomes. Correlations observed related to more extensive surgery, long-term intraocular tamponades and re-detachment. We identified that preoperatively pseudophakic patients (RRD eye or fellow eye) had higher scores on the vision specific mental health subscale than phakic patients. The mental health subscale consists of questions about troubling thoughts about the future and the eyesight. Perhaps, patients with a history of cataract extraction do not have to worry about undergoing a cataract extraction anymore.

We observed a significant correlation between post-operative BCVA and NEI VFQ-25 OCS and the subscale scores general vision, ocular pain, near activities, distance activities, mental health, role difficulties and driving. In contrast, Okamoto et al. did not find such a relationship [20]. This might have been due to differences in study design, since they included relatively younger patients with macula-on and macula-off RRD. This difference is underlined by the higher postoperative VA in the study by Okamoto et al. [20].

Generally, VA is considered as a major determinant of VR-QOL. This has been suggested in studies on macular hole, epiretinal membrane and diabetic retinopathy [34]–[35], [38]. Ophthalmologists consider post-operative expectations of VA when developing treatment strategies. However, VA may not always predict other aspects of visual function and NEI VFQ-25 scores are not always primarily correlated with VA, as was shown in previous studies on RRD [20].

In line with the study by Okamoto et al., we observed a significant correlation between contrast acuity and NEI VFQ-25 OCS [20]. In that study, the correlation between contrast acuity and the OCS differed between measurements with different types of charts, thus underlining the importance of the test method [20]. In a general population, reduced contrast sensitivity was associated with self-reported vision related disabilities, and it was associated with difficulties in performing tasks requiring distance judgments, such as night driving, and mobility [39]. This supports the correlation found in our study between diminished contrast acuity and a lower score on the NEI VFQ-25 subscores general vision, near activities, distance activities, vision related social functioning, vision related role difficulties, and driving.

Although we observed significant correlations between reduced CCI and VR-QOL parameters, we did not find any papers on a possible relation between both aspects. The observed correlations were small, and we therefore assume that unilateral, mild color vision defects have less impact on patients' well-being and visual functioning than unilateral defects in VA and contrast acuity. Also, it could be that the fellow eye compensates better for the defect in color vision in the affected eye than it does for the other two aspects of visual functioning.

Previously, we observed that both the duration and the height of macular detachment have a profound effect on the postoperative recovery of visual function, particularly BCVA and CCI [17]. In the present study, we observed that BCVA is highly correlated to VR-QOL. Even though we failed to demonstrate a direct correlation between the height of the macular detachment and NEI VFQ-25 scores, it might be clinically relevant to evaluate whether posturing of macula-off RRD patients would have a positive effect on postoperative BCVA and VR-QOL. The goal thereof would be to prevent a progression of a shallow detachment to a bullous one or to diminish submacular fluid in an already bullous one.

Our study highlights important aspects of the postoperative VR-QOL in macula-off RRD patients. Some limitations include a possible selection bias towards highly motivated patients, because they would have been more likely to have participated in this study. In addition, the studied population is modest. Our sample size is considered adequate for overall analyses [40], but it may be too limited for all subgroup analyses, resulting in an underreporting of possibly relevant associations. In addition, our analyses were performed on the postoperative visual function (BCVA, contrast acuity, saturated and desaturated CCI) in the RRD eye. The overall good visual function in the contralateral eye may have compensated for the defects in the RRD eye to a variable extent with regard to the different aspects of visual function. This may have mitigated the observed relations with VFQ outcomes to a variable extent.

At 12 months postoperatively, BCVA, contrast acuity, and CCI's in macula-off RRD eyes were still significantly worse for the operated eyes compared to their fellow control eyes. A lower VR-QOL (OCS and subscores) had the highest correlation with a worse postoperative BCVA and contrast acuity (in the RRD-eye). Although less pronounced, postoperative color vision disturbances (saturated and desaturated CCI) were significantly correlated with the NEI VFQ-25 OCS.
